# Morphological Detection and Functional Assessment of Regenerated Nerve after Neural Prosthesis with a PGLA Nerve Conduit

**DOI:** 10.1038/srep46403

**Published:** 2017-04-13

**Authors:** Dong Gao, Jun-Jian Jiang, Shi-Hui Gu, Jiu-Zhou Lu, Lei Xu

**Affiliations:** 1 Department of Hand Surgery, Huashan Hospital, Fudan University, Shanghai 200040, China; 2 Shanghai Key Laboratory of Forensic Medicine, Institute of Forensic Science, Ministry of Justice, Shanghai 200063, China

## Abstract

This study aimed to observe the morphological characteristics of a PGLA [poly(glycolide-co-L-lactide)] nerve conduit and regenerated nerve bundle in the human body using high-frequency ultrasound and examine functional recovery of the regenerated nerve using functional magnetic resonance imaging (fMRI) after neural prosthesis with a PGLA nerve conduit. Thirty-nine patients underwent high-frequency ultrasound, and one patient with superficial radial nerve injury (27-mm defect) underwent fMRI at one, three, and six postoperative months. The fMRI examination results were compared with sensory detection and high-frequency ultrasound results during the same follow-up window period. The normal and regenerated nerve bundles had similar ultrasonic imaging features. At one postoperative month, fMRI displayed activeness of the normal cortex in the brain region corresponding to the contralateral superficial radial nerve, while no activeness was observed on the ipsilateral side. From three to six postoperative months, fMRI revealed gradually increasing activeness in the brain region corresponding to the ipsilateral superficial radial nerve, but the activation area on the ipsilateral side was smaller than that on the contralateral side. Combining morphological detection of the regenerated nerve using high-frequency ultrasound and functional detection of the regenerated nerve using fMRI may be a valuable method for evaluating repair of peripheral nerve injury.

In 2010, Ray and Mackinnon^1^ proposed that laboratory and clinical evaluation methods following neural prosthesis are very important in various researches on artificial nerve conduits. At present, in clinical studies on artificial nerve conduits for the repair of nerve defects in the human body, recovery of nerve function is mainly assessed using clinical examination and electromyography (EMG)^1^. The former mainly includes detecting various sensory functions (monofilament tactility and two-point perception discrimination) and motor functions (muscle strength and kinds of evaluation scales). The latter mainly includes sensory/motor nerve conduction velocity, amplitude of sensory/motor action potential, spontaneous potential, and recruitment potential. Although all these indicators are directly linked to the nerve function, the main drawback is that the recovery of corresponding nerve function or electrophysiological variation can only be detected after a long time after the surgery. Thus, the doctors have to wait for a longer time after implantation of artificial nerve conduits to judge the actual effect of the implanted artificial nerve conduits. Moreover, 2 weeks, 1 month, and 3 months after the surgery are not only the key window periods to observe nerve regeneration and determine the effect of nerve conduits, but also the core periods for re-intervention. If the nerve conduits are found apparently collapsed in the early stage, it means that the nerve conduits are insufficient to support and protect the regenerated nerve, and the re-intervention measures should be applied as early as possible. Thus, early determination of nerve regeneration in the nerve conduits as well as dynamic variation of nerve conduits in the human body has a profound impact on the clinical studies of artificial nerve conduits. In addition, although clinical examination is most directly associated with the determination of nerve function, it may be prone to be affected by subjectivities of experimentalists and subjects. Meanwhile, although nerve EMG can be used to more objectively assess the nerve function recovery, it is not intuitive and easy to interpret and determine. Thus, finding a detection method that not only is more objective and intuitive, but also can determine the status of nerve regeneration in nerve conduits in the early stage, is of important practical value and significance for the clinical studies of artificial nerve conduits.

In previous animal experiments on artificial nerve conduits for the repair of nerve defects, various histological and immunological methods have been adopted to count regenerated nerve fibers, directly observe micro morphology, analyze morphology and wet weight of target muscles, and even analyze proteomics variation. These methods are objective and intuitive; they also enable to observe samples collected at any time point during the follow-up. Hence, they are excellent detection methods for determining the effectiveness of nerve conduits. However, these methods are not applicable to human clinical studies at all^2^. Currently, high-frequency ultrasound is the clinical method with the maximum potential value for directly observing peripheral nerve morphology. In 1988, Fornage^3^ introduced the feasibility of high-frequency ultrasound to examine peripheral nerves. From that time on, many studies have been conducted on the morphological characteristics of peripheral nerves using high-frequency ultrasound. The longitudinal section of nerve bundles presents fascicular, parallel, continuous high-echo bands with intermingled low-echo bands, and the cross-section presents a typical nested structure. The regenerated nerve should have similar morphology characteristics except the continuity between the proximal and distal nerve stumps. After neural prosthesis with PGLA nerve conduits, whether the status of conduits in high-frequency ultrasound tends to affect the observation of the regenerated nerve in the conduits as well as the detection accuracy for the anatomical structure of the regenerated nerve is still unknown and needs further exploration. Functional MRI (fMRI) is another commonly used method to explore the relationship between brain anatomy and human function, including the peripheral nerve function. When a human peripheral nerve is excited, the cerebral cortex is activated and leads to some hemodynamic changes, for example cerebral blood flow and increase in oxygen exchange, which in turn induce an increase in vascular oxygenated hemoglobin and a relative decrease in deoxygenated hemoglobin. In 1992, Kwong *et al*.^4^ studied the relationship between primary sensory nerves and fMRI imaging in brain. fMRI is considered one of the important tools in the nerve function researches owing to its advantages including noninvasiveness, no radioactivity, and good space and time resolution^5^. However, very few studies have been conducted on the regenerated nerve function after neural prosthesis with a PGLA nerve conduit. Therefore, this study used high-frequency ultrasound and fMRI techniques to explore the feasibility for determining the morphology and functional recovery of regenerated nerves after neural prosthesis with PGLA nerve conduits, with the objective to provide not only a method that can be used to timely, objectively, and intuitively determine nerve regeneration after neural prosthesis with PGLA nerve conduits, but also evidence for optimizing clinical evaluation strategies after neural prosthesis with nerve conduits.

## Materials and Methods

### Patients and grouping

Between June 2013 and December 2014, 39 patients with upper limb peripheral nerve defects, who received hand surgery in Huashan Hospital, Fudan University, were randomly selected. These patients underwent neural prosthesis with PGLA nerve conduits. All methods were carried out in accordance with relevant guidelines and regulations, and all experimental protocols were approved by Huashan Hospital, Fudan University.

The patients were aged 18–50 years (average 37 years). The inclusion criteria were as follows: (1) male or female; (2) had nerve defects in the forearm or hand after nerve injury or trimming nerve stoma, with a defect length ≤30 mm; (3) had a nerve injury for equal to or less than 6 months; (4) volunteered to participate in this clinical trial; were ready for active examination, treatment, and postoperative review; and signed informed consent. The exclusion criterion was as follows: had peripheral nerve–associated diseases (such as diabetes and alcoholism). The reasons for nerve injury mainly included incised wound caused by an electric saw, injury due to falling objects, mangling injury by heavy objects, and so forth. Time from injury to surgery: 24 patients received emergency one-stage repair within 8 h after injury, and 15 patients did not undergo emergency treatment but received 2-stage repair within 6 months after the injury.

Eleven patients suffered from superficial radial nerve injury in the forearm, while 28 cases had digital nerve injury, including 9 cases of thumb, 7 cases of index finger, 3 cases of middle finger, and 2 cases of little finger. All the patients had accompanying sensory disturbances in the corresponding innervated area before the surgery. After the nerve stomas were trimmed intraoperatively, the defect length ranged 5–27 mm, with a mean length of 19.3 mm.

In this study, the artificial PGLA nerve conduits were produced by Shanghai Tianqing Co., Ltd., based on Donghua University patented technologies of controlling the cross-linking property of polymeric materials and three-dimensional electrostatic spinning braiding. The conduits were officially approved for clinical trials by the China Food and Drug Administration. The PGLA nerve conduits were made of poly (glycolide-*co*-L-lactide), and had a length of 10–50 mm and an inner diameter of 3 mm. An appropriate model was selected according to the defect length of the injured nerve measured intraoperatively.

## Methods

### Surgical treatment of injured nerves

The proximal and distal stomas of the injured nerves were trimmed to expose the normal nerve papilla. The defect length and nerve diameter were measured. An appropriate model of a PGLA nerve conduit was selected for end-to-end anastomosis using 8–0 prolene atraumatic sutures, where the suture at the epineurium was 2 mm away from the nerve stoma. After anastomosis, it should be ensured that 2 mm of both proximal and distal ends were in the nerve conduits, with no tension at all. All the anastomosis surgeries were completed by the same senior surgeon in the hospital.

### Postoperative detection

#### High-frequency ultrasound

The characteristics of a nerve conduit and regenerated nerve were observed at one, three, and six postoperative months using the high-frequency ultrasound (Philips iU-22 ultrasound, Philips L17–5 high-frequency probe, Royal Philips, the Netherlands).

Using cross-sectional and longitudinal sectional dynamic high-frequency ultrasound, the cross-sectional areas of the normal and regenerated nerves could be measured during various postoperative periods.

#### Functional magnetic resonance imaging

One patient with the greatest defect (27 mm) on the superficial radial nerve underwent fMRI examination of the innervated area of the superficial radial nerve as well as sensory function and electrophysiological examinations on the ipsilateral and contralateral sides.

The fMRI examinations were completed in the imaging center using 3.0-T MRI system (Siemens, Germany). Chip stimulating electrodes were placed in the thumb–index webs of both hands, and then the patients underwent a head MRI scan separately under the conventional resting state and under electrically stimulating thumb–index webs. The data were automatically analyzed using fMRI post-processing software (Siemens, Germany) in the MRI instrument to obtain contrast cerebral functional images between stimulating and resting states. Five times of sampling data at echo-planar imaging (EPI) commencement were excluded to decrease the impact of hemodynamics on signals in activated areas and avoid magnetic saturation effect. Then, the cerebral functional images were superimposed on the corresponding T1WI anatomies.

#### Other functional examinations

The sensory function examination included cutaneous sensation in the ipsilateral and contralateral innervated areas, which could be detected using a Baseline tactile monofilament evaluator (FEI, USA) and a static two-point perception discrimination detector (Jamar, USA). In terms of nerve electrophysiological examination, sensory nerve conductions of superficial radial nerves in bilateral limbs were tested using Keypoint nerve EMG equipment (Medtronic, Denmark), including sensory nerve conduction velocity and amplitude of SNAP wave.

## Results

### PGLA nerve conduits and regenerated nerves detected by ultrasound

**Figure 1:**
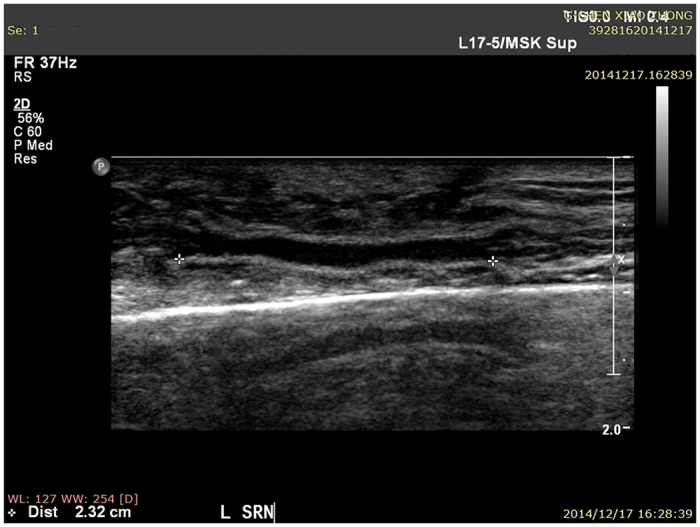
PGLA nerve conduit and regenerated superficial radial nerve in high-frequency ultrasound at one postoperative month.

**Figure 2:**
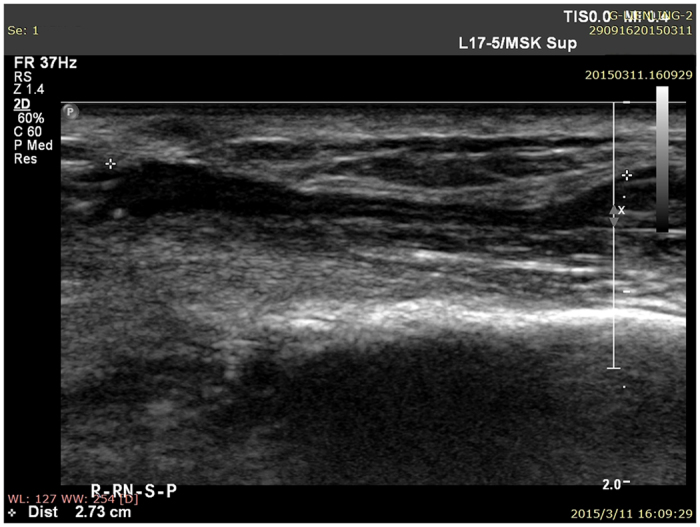
Regenerated superficial radial nerve in high-frequency ultrasound at three postoperative months.

**Figure 3:**
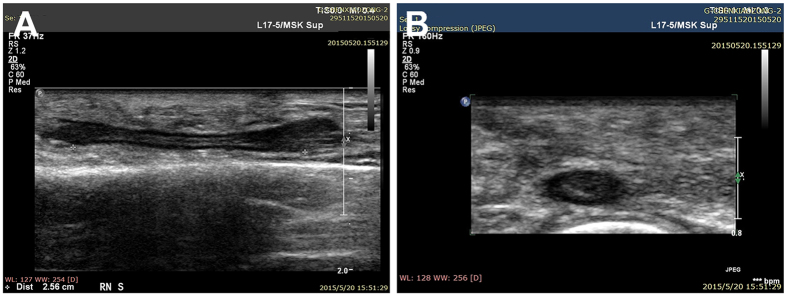
Regenerated superficial radial nerve in high-frequency ultrasound at six postoperative months. (**A**) Longitudinal section; (**B**) transverse section.

**Figure 4:**
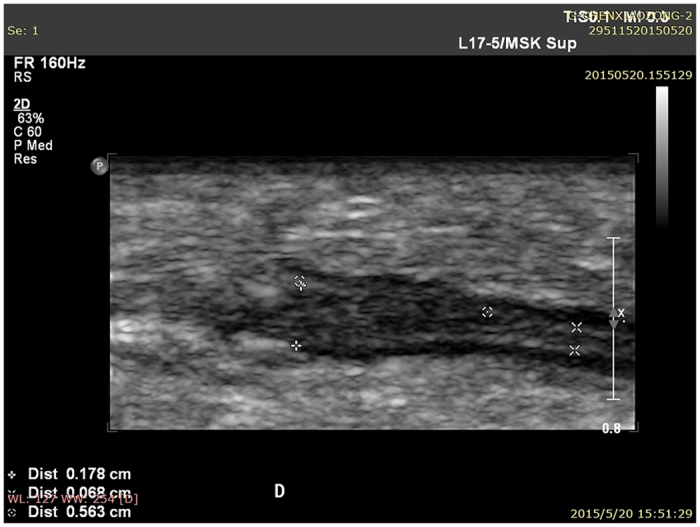
The distal anastomosis features of the regenerated nerve in high-frequency ultrasound.

When high-frequency ultrasound was used to test PGLA nerve conduits in the human body at one postoperative month, the conduits presented black and low-density regions, and still no disconnection was observed between the regenerated superficial radial nerve and the distal stoma (Fig. 1). At three and six postoperative months, the normal and regenerated nerve bundles had similar ultrasonic imaging features, where the cross-sectional nerve bundles presented a typical nested structure (Figs 2 and 3). However, the longitudinal section of the nerve bundles presented fascicular, parallel, incompletely continuous high-echo bands with intermingled low-echo bands. During the early postoperative period, although the regenerated nerves had a small diameter, they could still be clearly seen in high-frequency ultrasound (Fig. 2). Also, the high-frequency ultrasound could also clearly display the distal anastomosis features of the regenerated nerve (Fig. 4).

**Figure 5:**
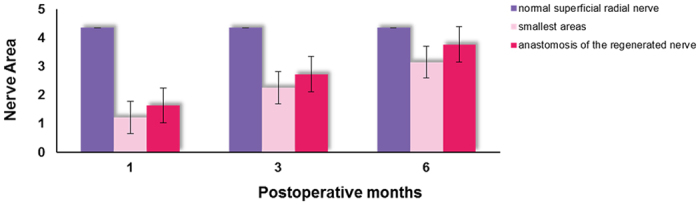
Areas of the superficial radial nerve during different periods after PGLA nerve conduit repair.

The area of the normal superficial radial nerve was found to be 4.35 ± 0.23 mm^2^, while the smallest areas/areas at the anastomosis of the regenerated nerve at one, three, and six postoperative months were 1.22 ± 0.15 mm^2^/1.64 ± 0.20 mm^2^, 2.26 ± 0.31 mm^2^/2.73 ± 0.25 mm^2^, and 3.15 ± 0.23 mm^2^/3.77 ± 0.18 mm^2^, respectively. Figure 5 shows areas of the normal and regenerated nerves during different periods after repair of the superficial radial nerve with PGLA nerve conduits. The changes in the areas of the regenerated nerves were the same as shown in Figs 2 and 3. The superficial radial nerve regenerated from the proximal stoma to the distal end had the smallest diameter in the middle of the track which increased at the proximal junction and distal anastomosis. Meanwhile, at 1, 3, and 6 months after the surgery, the areas in the middle part as well as distal anastomosis of the regenerated nerve gradually increased, and were close to the area of the normal nerve at six postoperative months.

**Figure 6:**
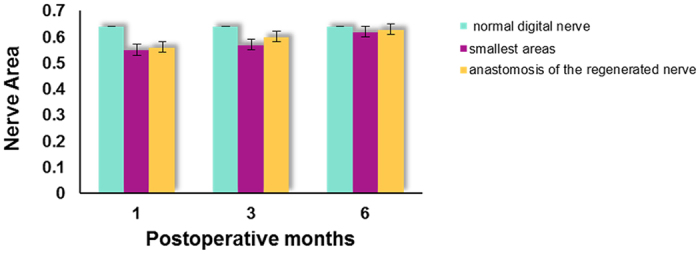
Areas of the digital nerve during different periods after PGLA nerve conduit repair.

The area of the normal digital nerve was found to be 0.64 ± 0.21 mm^2^, while the smallest areas/areas at anastomosis of the regenerated nerve at one, three, and six postoperative months were, respectively, 0.55 ± 0.14 mm^2^/0.56 ± 0.17 mm^2^, 0.57 ± 0.20 mm^2^/0.60 ± 0.15 mm^2^, and 0.62 ± 0.22 mm^2^/0.63 ± 0.17 mm^2^. Figure 6 shows areas of the digital nerve during different periods after PGLA nerve conduit repair. The area of regenerated digital nerve was close to that of the normal nerve regardless of the middle part or distal anastomosis, which did not show a statistically significant difference compared with the area of the normal nerve. Also, the area of the regenerated nerve gradually increased with time so that it was comparable to that of the normal nerve.

### fMRI results and comparison with other detection indicators

**Figure 7:**
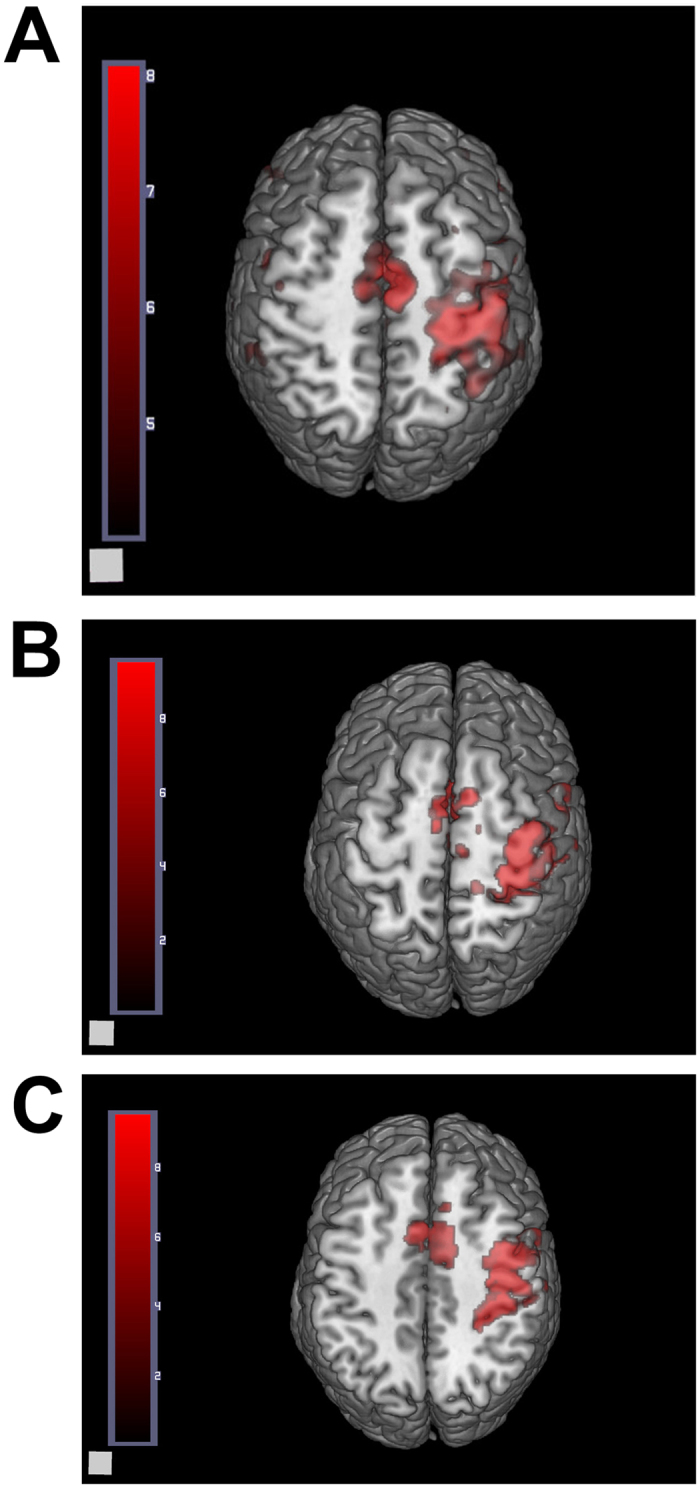
Activeness of the normal cortex in the brain region corresponding to the contralateral superficial radial nerve after different periods during fMRI examination. (**A**) One postoperative month; (**B**) three postoperative months; (**C**) six postoperative months.

**Figure 8:**
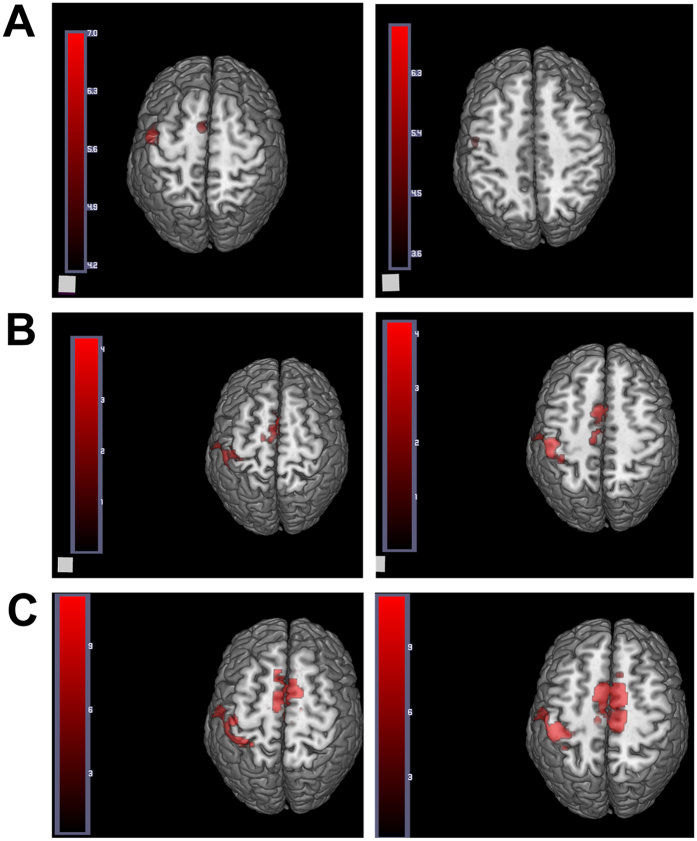
Activeness of the cortex in the brain region corresponding to the ipsilateral superficial radial nerve after different periods during fMRI examination. (**A**) One postoperative month; (**B**) three postoperative months; (**C**) six postoperative months.

At one postoperative month, fMRI displayed activeness of the normal cortex in the brain region corresponding to the contralateral superficial radial nerve (Fig. 7A), and at three and six postoperative months (Fig. 7B and C), they displayed similar active anatomical location and activeness extent in the brain region corresponding to the contralateral superficial radial nerve. At one postoperative month, no activeness of the cortex in the brain region corresponding to the ipsilateral superficial radial nerve was noted (Fig. 8A). Meanwhile, the high-frequency ultrasound demonstrated a disconnection between the regenerated superficial radial nerve and the distal stoma (Fig. 1). At this time, patients mainly complained of significant numbness, monofilament tactility, and two-point perception discrimination at the superficial radial nerve–dominated region, and the electrophysiological examination prompted the absence of a definite sensory nerve action potential (SNAP) wave.

At three postoperative months, fMRI revealed some activeness in the brain region corresponding to the ipsilateral superficial radial nerve (Fig. 7B). The high-frequency ultrasound showed that the regenerated superficial radial nerve was already connected with the distal stoma, but had a small diameter (Fig. 2). Also, the monofilament tactility and two-point perception discrimination were restored in the superficial radial nerve–dominated area; the monofilament tactility reached 4.56 (2.83 on the contralateral side), and the two-point perception discrimination was 10 mm (3 mm on the contralateral side). The electrophysiological examination displayed a visible SNAP wave (velocity 14.5 m/s, amplitude 1.8 μV).

At six postoperative months, fMRI revealed more activeness (Fig. 7C) in the brain region corresponding to the ipsilateral superficial radial nerve than before. High-frequency ultrasound demonstrated that the diameter of the regenerated superficial radial nerve was larger compared with that at three postoperative months, but it was still smaller than that of the normal nerve (Fig. 3). Also, the monofilament tactility and two-point perception discrimination were well restored in the superficial radial nerve–dominated area; the monofilament tactility was up to 3.61 (2.83 on the contralateral side) and two-point perception discrimination was up to 5 mm (3 mm on the contralateral side). Meanwhile, the electrophysiological examination displayed a visible SNAP wave (velocity 25.7 m/s, amplitude 7.1 μV).

## Discussion

Nerve function recovery is mainly used to reflect the clinical application value of artificial nerve conduits in clinical studies on the repair of nerve defects with artificial nerve conduits in the human body. Evaluation of nerve function recovery mainly depends on conventional and routine measures such as clinical examination and electrophysiology^1^, while morphological variation of regenerated nerves after neural prosthesis with artificial nerve conduits has been less studied. It is believed that compared with the evaluation of nerve function, studies on the morphology of a regenerated nerve allow clinicians to determine the growth of the regenerated nerve in advance, which is conducive to judging and intervening nerve regeneration in the early stage after neural prosthesis with nerve conduits. Also, traditional evaluation methods for nerve functions are prone to be affected by subjectivities of experimentalists and subjects, adversely affecting the credibility of the findings^2^.

High-frequency ultrasound is the most widely used method to directly observe the morphology of the peripheral nerve^6^. High-frequency ultrasound is noninvasive and easily accepted by patients, which is beneficial for multiple dynamic follow-ups after repair of the peripheral nerve. It is an important means for studying morphological variations after peripheral nerve injury. With the emergence of higher-frequency linear-array probes and development of image post-processing technology, high-frequency ultrasound detection can obtain clear and precise peripheral nerve images, which can not only display distribution, traveling, and thickness of the peripheral nerve as well as its anatomic relationship with the peripheral tissues^7^, but also reveal bundle structures inside the nerve. Thus, high-frequency ultrasound can be used to evaluate the continuity of an injured nerve after direct anastomosis^8^,^9^ and determine conduit degradation after repair of peripheral nerve defects with nerve conduits^10^. Therefore, it is feasible to apply high-frequency ultrasound to detect the morphology of a regenerated nerve after repair of the peripheral nerve defects with PGLA nerve conduits. In this study, all patients were followed up during multiple postoperative detection window periods after neural prosthesis with PGLA nerve conduits. It was found that PGLA nerve conduits presented a low-density shadow in high-frequency ultrasound images. Meanwhile, the normal and regenerated nerve bundles had similar ultrasound imaging features: the cross-sectional images presented a typical nested structure, while the longitudinal sectional images presented fascicular, parallel, incompletely continuous high-echo bands with intermingled low-echo bands, which were consistent with high-frequency ultrasound results of the normal nerve. Therefore, high-frequency ultrasound was able to position PGLA nerve conduits and the inside nerve structures. Figures 1, 2 and 3 reveal dynamic features during different time periods after repair of the superficial nerve defects. They clearly show gradual growth of the regenerated nerve from the proximal stoma to the distal stoma, and an increase in diameter. Therefore, high-frequency ultrasound could be used to dynamically monitor features in PGLA nerve conduits, thereby allowing to timely understand whether implanted PGLA nerve conduits could provide long-term and sufficient protection to the regenerated nerve, which was consistent with the results in the literature^10^. Thus, it is believed that high-frequency ultrasound is appropriate for morphological detection during different periods after neural prosthesis with PGLA nerve conduits. It has a very high value and utility for clinical studies on the repair of peripheral nerve defects with PGLA nerve conduits.

Although high-frequency ultrasound can reflect morphological variations of the regenerated nerve, morphological continuity in ultrasound images does not necessarily mean the effective connection of the regenerated nerve (due to the limitation of ultrasound resolution,), not to mention the recovery of nerve function. This means that the morphological continuity in high-frequency ultrasound is not closely associated with the recovery extent of nerve function. Thus, an objective method needs to be developed to evaluate regenerated nerve function during nerve regeneration. At present, MRI is another important technique for the morphological detection of the peripheral nerve. With the rapid development of MRI devices and digital post-processing techniques in recent years, clinical studies on examining peripheral nerve injury with MRI have gained attention^11^. Gustav Andreisek *et al*.^12^ compared image features between three normal and injured nerves in the forearm, and found that T1-weighted spin-echo sequences could clearly display part of anatomic details of the normal nerve. For example, a normal nerve mostly has a smooth, round, or oval structure, its signal intensity is similar to that of the muscles, and it is often surrounded by high signals generated by the epineurium. Meanwhile, T2-weighted images based on nerve and its dominated muscles are different from short-time inversion recovery signal intensity and have different features at different time points, which allows MRI to differentiate between axonal fracture and nerve separation^13^. Skorpil^14^ applied a new MRI technique, diffusion direction–dependent imaging (DDI), after processing to isolate human sciatic nerve images from surrounding tissue images, to clearly display nerve morphology. In 2012, Zhou *et al*.^15^ used the diffusion tensor imaging technique to examine median and ulnar nerves in the human forearm, which enabled to display a nerve in the human forearm with a diameter of about 3 mm. However, it could only reveal nerve continuity or presence of injury and other changes, and was still unable to accurately observe and measure the fine structure inside the nerve. Bilegen *et al*.^16^ successfully obtained fine imaging of the human median nerve using MRI with a field intensity of 9.4 T. The aforementioned results reflect very good objectivity and visualization ability of MRI in displaying the morphology of the peripheral nerve. It should be of significant value if MRI can be applied in accurately observing a regenerated nerve after neural prosthesis with nerve conduits in humans. Of course, for a field intensity of the current MRI device, 3.0 T, MRI is unable to effectively display the fine anatomic structure of most peripheral nerves in the human body. However, even if a field intensity of 7.0 9.0 T is obtainable using an experimental MRI instrument, the instrument is not commercially available and expected to be very costly. Hence, it is not suitable for continuous and dynamic follow-up after repair of the peripheral nerve. Appling the MRI technique in the morphological detection of the peripheral nerve is temporarily unable to meet the clinical needs, but fMRI, which has rapidly developed in recent years, may be used for detecting regenerated nerve function. At present, the mainstream measure of fMRI is blood oxygen level–dependent (BOLD) fMRI. It is an MRI technique based on the magneto-sensitive effect of deoxygenated hemoglobin. If appropriate stimulation is applied to limb peripheral effectors, it can be transmitted into the brain by nerves to excite corresponding brain regions, which increases metabolism of excited regions and local blood flow, thereby increasing oxyhemoglobin content in the excited brain region. However, the nonexcited brain regions are dominated by deoxygenated hemoglobin, which is paramagnetic. BOLD-fMRI adopts EPI sequences for scanning (T2*WI), where a shortened T2 decreases tissue signals in nonexcited brain regions, thereby highlighting relatively high signals in the excited brain regions (T2*WI). After processing, brain regions with varying degrees of excitation may present different colors, thereby displaying the performance of cerebral function. Compared with other imaging methods of cerebral function, the BOLD method has a higher spatial resolution, higher temporal resolution, absence of electromagnetic radiation, and lower cost. Furthermore, fMRI allows repeated, longitudinal, and large-sample studies. Thus, it is a functional measure suitable for continuous, dynamic, and early detection after repair of peripheral nerve injury. In this study, the BOLD-fMRI technique was used to perform continuous and dynamical detection after repair of superficial radial nerve with PGLA nerve conduits. The results showed that with the increasing recovery time, a dynamic variation of activeness in cortex appeared, dominated by the ipsilateral superficial radial nerve. Meanwhile, the dynamic variation of fMRI was associated with the morphological dynamic variation of regenerated nerves and dynamic variation of sensory function indicators (monofilament tactility, static two-point perception discrimination, and electrophysiological examination). Especially, at three postoperative months, high-frequency ultrasound revealed that the regenerated superficial radial nerve already reached the distal stoma, but had a very small diameter in the middle part. At this time, fMRI results indicated that stimulating the ipsilateral superficial radial nerve–dominated area would induce activeness variation of cortex in the corresponding brain region, which effectively proved that although only some continuity was restored in the regenerated nerve, objective, definite, and intuitive functional recovery occurred. The results strongly suggested good quality of the regenerated nerve. Therefore, it is believed that the fMRI technique can be used to early, dynamically, objectively, and intuitively determine the functional recovery of the regenerated nerve. Combining morphological detection of the regenerated nerve using high-frequency ultrasound and functional detection of the regenerated nerve using fMRI tends to provide a valuable evaluation method for repairing peripheral nerve injury. In addition, although fMRI was found to be associated with sensory function recovery indicators (monofilament tactility, static two-point perception discrimination, and electrophysiological examination) and morphological continuity of the regenerated nerve using high-frequency ultrasound, the sample size in this study was small. Thus, it is necessary to perform in-depth studies involving more clinical cases.

## Additional Information

**How to cite this article**: Gao, D. *et al*. Morphological Detection and Functional Assessment of Regenerated Nerve after Neural Prosthesis with a PGLA Nerve Conduit. *Sci. Rep.* **7**, 46403; doi: 10.1038/srep46403 (2017).

**Publisher's note:** Springer Nature remains neutral with regard to jurisdictional claims in published maps and institutional affiliations.
